# Quercetin enhances fatty acid β-oxidation by inducing lipophagy in AML12 hepatocytes

**DOI:** 10.1016/j.heliyon.2021.e07324

**Published:** 2021-06-18

**Authors:** Misato Fukaya, Yoriko Sato, Shinji Kondo, Shin-ichi Adachi, Fumiaki Yoshizawa, Yusuke Sato

**Affiliations:** a Department of Agrobiology and Bioresources, School of Agriculture, Utsunomiya University, Tochigi, 3218505, Japan; b Center for Bioscience Research and Education, Utsunomiya University, Tochigi, 3218505, Japan; c Department of Animal Science, School of Agriculture, Tokai University, Kumamoto, 8628652, Japan

**Keywords:** Quercetin, Lipophagy, Fatty acid β-oxidation, Hepatocyte, AMPK

## Abstract

Recent evidence demonstrated that chronic intake of quercetin attenuated hepatic fat accumulation in various animal models of obesity and diabetes. However, whether quercetin has the ability to enhance energy metabolism in hepatocytes and its exact mechanisms have yet to be identified. In the present study, we investigated whether quercetin directly enhanced the energy metabolism of cultured hepatocytes by focusing on lipophagy, involving selective autophagic degradation of lipid droplets. As an indicator of mitochondrial respiration, oxygen consumption was measured following 12-h treatment with quercetin or its related flavonoids, isorhamnetin and rutin (10 μM) using an extracellular flux analyzer. Treatment of alpha mouse liver 12 (AML12) hepatocytes with quercetin enhanced mitochondrial respiration, but isorhamnetin and rutin did not. Results of a palmitate-bovine serum albumin fatty acid oxidation assay showed that quercetin significantly increased the oxygen consumption of AML12 hepatocytes, suggesting enhanced fatty acid β-oxidation. However, as expression levels of mitochondrial oxidative phosphorylation proteins were unaltered by quercetin, we explored whether lipophagy contributed to enhanced fatty acid β-oxidation. Increased colocalization of lipid droplets and lysosomes confirmed that quercetin promoted lipophagy in AML12 hepatocytes. Furthermore, pharmacological inhibition of the autophagy–lysosomal pathway abolished the enhancement of fatty acid β-oxidation induced by quercetin in AML12 hepatocytes, suggesting that the enhancement of lipophagy by quercetin contributed to increased fatty acid β-oxidation. Finally, we showed that quercetin could activate AMPK signaling, which regulates autophagy even under nutrient-sufficient conditions. Our findings indicate that quercetin enhanced energy metabolism by a potentially novel mechanism involving promotion of lipophagy to produce the substrate for fatty acid β-oxidation in mitochondria through activation of AMPK signaling. Our results suggest the possibility that nutrient-induced lipophagy might contributes to the reduction of fat in hepatocytes.

## 1 Introduction

Flavonoids have been demonstrated to prevent various lifestyle-related diseases as dietary supplements [1]. Quercetin, one of the most prominent flavonoids present in fruits, vegetables, and tea, acts as an antioxidant *in vitro* and *in vivo* [1, 2, 3, 4]. Numerous reports have demonstrated the beneficial effect of quercetin on obesity, metabolic syndromes, and cardiovascular disorders using humans, animal models, and cultured cells. Indeed, some human studies have been performed to evaluate the effects of quercetin on obesity. As a result, intake of 100 mg/day of quercetin or quercetin-rich onion extract for 12 weeks decreased the total body fat and body mass index of overweight individuals and obese female university students, respectively [5, 6]. Moreover, in animal models of obesity, such as streptozotocin-induced diabetic mice and diet-induced obesity mice, quercetin supplementation improved metabolic abnormalities [7, 8]. Chronic supplementation with 0.05% quercetin suppressed fat accumulation and inflammation in the liver and visceral white adipose tissue of mice fed a Western diet [9, 10]. In other disease models, quercetin ameliorated nonalcoholic fatty liver disease (NAFLD) and alcoholic liver disease (ALD) by reducing accumulation of hepatic lipid droplets [11, 12, 13]. The beneficial effects of quercetin are likely caused by upregulated expression of genes related to lipid metabolism, mitochondrial biogenesis, and their accompanying transcriptional regulators [8, 9, 10]. Indeed, previous reports have shown that quercetin might directly affect the activities of some enzymes, as well as expression of heat shock proteins and cellular signaling. In human hepatocytes, quercetin attenuated oxidative stress induced by ethanol exposure by upregulating heme oxygenase-1 expression through activation of p38 and extracellular signal-regulated kinase (ERK) signaling, and subsequent nuclear translocation of nuclear factor E2-related factor 2 [14, 15, 16]. Although quercetin reportedly exerts hepatoprotective effects, it remains unknown whether it has the ability to enhance energy metabolism in hepatocytes. Importantly, recent reports demonstrated that quercetin ameliorated high-fat-diet-induced NAFLD *in vivo* by promoting lipophagy, which involves selective autophagic degradation of lipid droplets [17, 18]. Recent reports suggest that quercetin activates autophagy through mechanistic target of rapamycin (mTOR) or signal transducer and activator of transcription 3 signaling in gastric cancer, ovarian cancer, and effusion lymphoma cells [19, 20, 21]. As mTOR is a key regulator of autophagy and regulates lipid metabolism in the liver [22, 23], we hypothesized that quercetin-mediated reduction of lipid droplets in hepatocytes is related to enhanced lipophagy through the inactivation/activation of certain intracellular signaling pathways.

In this study, we investigated whether quercetin and its related flavonoids (isorhamnetin and rutin) enhance energy metabolism (glycolysis and mitochondrial respiration) in alpha mouse liver 12 (AML12) hepatocytes. Rutin is a quercetin glycoside in which the disaccharide β-rutinose is glycosidically bonded to the 3-position of quercetin, whereas isorhamnetin is a derivative of quercetin and one of its metabolites. Similar to quercetin, neither rutin or isorhamnetin have been investigated for their effects on energy metabolism. To explain the mechanism by which quercetin enhanced energy metabolism, we hypothesized that quercetin induced lipophagy and autophagy of lipid droplets in hepatocytes. Our results provide evidence of enhanced energy metabolism in AML12 hepatocytes following quercetin treatment, as well as its mechanism. Moreover, our findings are the first to suggest that lipophagy induced by nutrients, such as flavonoids, contributes to the reduction of fat in hepatocytes.

## 2 Materials and methods

### 2.1 Cell culture and induction of lipid droplet accumulation

AML12 hepatocytes purchased from American Type Culture Collection (Manassas, VA, USA) were cultured in Dulbecco's Modified Eagle's Medium with Nutrient Mixture F-12 (DMEM/F-12) supplemented with 10% fetal bovine serum (FBS), 5 μg/mL human insulin, 5 μg/ml transferrin, 3 ng/ml selenium, 40 ng/ml dexamethasone, 100 U/ml penicillin, and 100 μg/ml streptomycin at 37 °C with 5% CO_2_. Cells were seeded on six- or twelve-well microplates for protein and gene expression analyses, chamber slides for staining, and XFp microplates for metabolic measurements. All cells used in this study were exposed to DMEM containing 250 μM palmitic acid, 500 μM oleic acid, and 1% fatty acid free-bovine serum albumin (BSA) for 24 h to induce lipid accumulation.

### 2.2 RNA extraction and quantitative real-time PCR (qRT-PCR)

Expression of target and reference genes was measured using qRT-PCR, with *Gapdh* as the reference gene. Total RNA was isolated from cultured AML12 hepatocytes according to a standard TRIzol–chloroform protocol. cDNA was synthesized from 1 μg of total RNA using iScript reverse transcriptase (Bio-Rad, Hercules, CA, USA), and qRT-PCR was performed using a MyiQ2 real-time PCR system (Bio-Rad). Primer sets were designed by PrimerBank or Primer3 software. Primer sequences were as follows: *Tfam* forward, GAGGCAAAGGATGATTCGGCTC; *Tfam* reverse, CGAATCCTATCATCTTTAGCAAGC; *Ppara* forward, CTGCAGAGCAACCATCCAGAT; *Ppara* reverse, GCCGAAGGTCCACCATTTT; *Pparg* forward, CACAATGCCATCAGGTTTGG; *Pparg* reverse, GCTGGTCGATATCACTGGAGATC; *Ppargc1a* forward, ATGTGTCGCCTTCTTGCTCT; *Ppargc1a* reverse, ATCTACTGCCTGGGGACCTT; *Cpt1* forward, CTCCGCCTGAGCCATGAAG; *Cpt1* reverse, CACCAGTGATGATGCCATTCT; *Cpt2* forward, CAGCACAGCATCGTACCCA; *Cpt2* reverse, TCCCAATGCCGTTCTCAAAAT; *Map1lc3b* forward, CGATACAAGGGGGAGAAGCA; *Map1lc3b* reverse, ACTTCGGAGATGGGAGTGGA; *Plin2* forward, GACCTTGTGTCCTCCGCTTAT; *Plin2* reverse, CAACCGCAATTTGTGGCTC; *Gapdh* forward, CATCACTGCCACCCAGAAGACTG; *Gapdh* reverse, ATGCCAGTGAGCTTCCCGTTCAG.

### 2.3 Protein extraction and immunoblot analyses

Proteins were extracted from cultured AML12 hepatocytes and homogenized in sodium dodecyl sulfate sample buffer containing 125 mM Tris–HCl (pH 6.8), 5% β-mercaptoethanol, 2% sodium dodecyl sulfate, and 10% glycerol. Extracted proteins were separated on acrylamide gels, transferred onto polyvinylidene fluoride membranes (GE Healthcare, Chicago, IL, USA), and blocked using 5% BSA. A ChemiDoc XRS Imager (Bio-Rad) was used to visualize bands. The following antibodies were used at a dilution of 1:1000 for immunoblot analysis: anti-total OXPHOS (Cat. No. ab110413; Abcam, Cambridge, UK), anti-LC3B [Cat. No. 2775; Cell Signaling Technology (CST), Danvers, MA, USA], anti-phospho-ERK1/2 (Thr202/Tyr204) (Cat. No. 9101, CST), anti-total-ERK1/2 (Cat. No. 9102, CST), anti-phospho-Akt (Ser473) (Cat. No. 4060S, CST), anti-total-Akt (Cat. No. 9272S, CST), anti-phospho-mTOR (Ser2448) (Cat. No. 2971, CST), anti-total-mTOR (Cat. No. 2983P, CST), anti-phospho-AMPKα (Thr172) (Cat. No. 2535, CST), and anti-total-AMPKα (Cat. No. 2532, CST). GAPDH (1:2000; Cat. No. 6C5; Santa Cruz Biotechnology, Dallas, TX, USA) was used as an internal standard.

### 2.4 Fluorescent staining

To visualize lipophagy induced by quercetin, AML12 hepatocytes were stained with BODIPY 493/503 (Sigma-Aldrich, St. Louis, MO, USA) for lipid droplets and LysoTracker Red DND-99 (Molecular Probes, Eugene, OR, USA) for lysosomes. Briefly, living cells were exposed to LysoTracker Red for 2 h before fixation with 4% paraformaldehyde for 15 min, followed by staining with BODIPY 493/503 for 30 min. Fluorescent images were obtained with a Leica DM5000B microscope (Wetzlar, Germany). The whole images of each well (n = 7) were analyzed by using the ImageJ plugins Colocalization_Test to evaluate colocalization of BODIPY and Lysotracker in hepatocytes (https://imagej.nih.gov/). Evaluation of colocalization was expressed by Rtotal (Pearson's correlation coefficient for the whole image, which varies between −1 and 1) divide by total BODIPY intensity.

### 2.5 Energy metabolism measurement

To measure energy metabolism, a Seahorse XFp Extracellular Flux Analyzer (Agilent Technologies, Santa Clara, CA, USA) was used to detect the oxygen consumption rate (OCR) and extracellular acidification rate. Glycolytic capacity was determined by Seahorse XF Glycolysis Stress Test (Agilent Technologies) according to the manufacturer's protocol. Briefly, cells were incubated in glucose and pyruvate-free XF base media for 1 h, followed by sequential injection of glucose, oligomycin, and 2-deoxy-D-glucose (2DG) onto the culture plate to determine basal glycolytic capacity, maximum glycolytic capacity, and glycolytic reserve, respectively. Mitochondrial respiration capacity was determined using a Seahorse XF Cell Mito Stress Test kit according to the manufacturer's protocol. Briefly, cells were incubated in medium containing 10 mM glucose, 1 mM pyruvate, and 2 mM glutamate for 1 h, followed by sequential injection of oligomycin, carbonyl cyanide-4-(trifluoromethoxy) phenylhydrazone (FCCP), and rotenone with antimycin A to determine non-mitochondrial oxygen consumption, basal respiration, maximum respiration, H^+^ leak, ATP production, and spare respiration capacity.

### 2.6 Fatty acid oxidation (FAO) assay

An FAO assay was performed in combination with the Cell Mito Stress Test. First, cells were cultured in XF base media containing 0.5 mM glucose, 1 mM glutamine, 500 μM carnitine (required to transport long chain fatty acid into mitochondria), and 1% FBS overnight. The following day, cells were incubated in Krebs-Henseleit buffer supplemented with 5 mM HEPES, 2.5 mM glucose, 500 μM carnitine, and 170 μM fatty acid palmitate-BSA for 1 h before commencing measurements.

### 2.7 Measurement of triglycerides

Triglyceride levels in AML12 hepatocytes were measured by E-Test Wako (Wako Pure Chemistry Industries, Osaka, Japan) according to the manufacturer's protocol and normalized to the protein concentration.

### 2.8 Statistical analysis

All data are presented as mean ± SEM. P values less than 0.05 were considered significant and all assessments of significance were performed with Student's t-test.

## 3 Results

### 3.1 Quercetin enhanced energy metabolism (glycolysis and mitochondrial respiration) in AML12 hepatocytes

**Figure 1:**
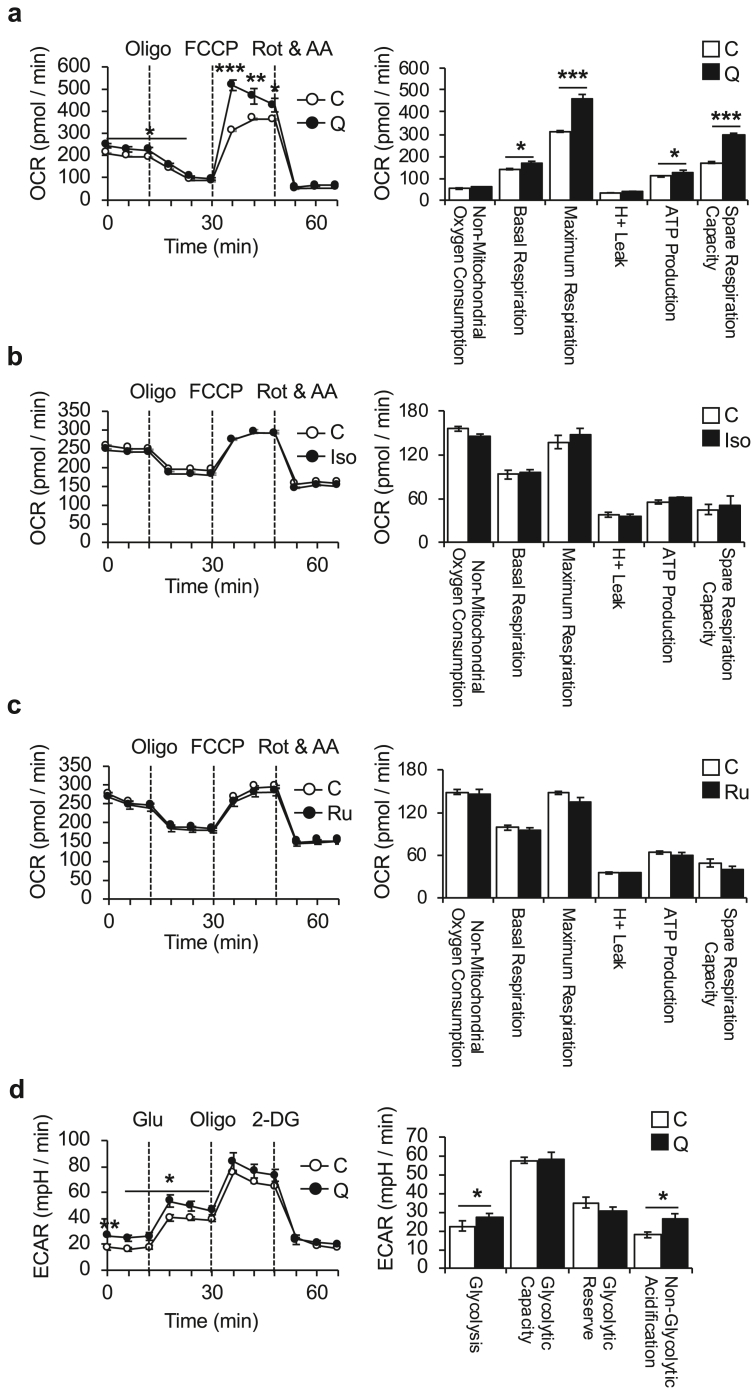
Effect of quercetin, isorhamnetin, and rutin on mitochondrial respiration of AML12 hepatocytes. A Cell Mito Stress Test was performed to investigate the effect of (a) quercetin, (b) isorhamnetin, and (c) rutin on mitochondrial respiration. Oligomycin (Oligo), carbonyl cyanide-p-trifluoromethoxyphenylhydrazone (FCCP), and rotenone with antimycin A (Rot & AA) were sequentially injected onto culture microplates to determine individual parameters of mitochondrial function (non-mitochondrial oxygen consumption, basal respiration, maximum respiration, H^+^ leak, ATP production, and spare respiration capacity). The results show enhanced mitochondrial respiration following quercetin treatment (10 μM) for 12 h. Significance was determined with a two-tailed Student's t-test (∗p < 0.05, ∗∗p < 0.01, ∗∗∗p < 0.001). Values represent mean ± SEM (*n* = 3). C, control; Iso, isorhamnetin; Q, quercetin; R, rutin. (d) Results of Glycolysis Stress Test show enhanced glycolysis following quercetin treatment (10 μM) for 12 h. Glucose (Glu), Oligomycin (Oligo), and 2-deoxy-D-glucose (2-DG) were sequentially injected onto culture microplates to determine individual parameters of glycolysis (glycolysis, glycolytic capacity, glycolytic reserve, and non-glycolytic acidification).

To obtain direct evidence of whether flavonoids including quercetin, isorhamnetin, and rutin enhanced energy metabolism in hepatocytes, we performed Mito Stress and Glycolysis Stress Tests using an extracellular flux analyzer. As an indicator of mitochondrial respiration, OCR was measured 12 h after treatment with flavonoids (10 μM). Compared with controls, quercetin significantly increased the OCR, both under basal and FCCP-treated conditions, but neither isorhamnetin or rutin affected the OCR (Figure 1a–c). Furthermore, quercetin partially increased the basal glycolysis of AML12 hepatocytes compared with the control (Figure 1d). These data demonstrate, for the first time, that quercetin can enhance the energy metabolism of cultured AML12 hepatocytes.

### 3.2 Quercetin enhanced energy metabolism by activating fatty acid β-oxidation in AML12 hepatocytes

**Figure 2:**
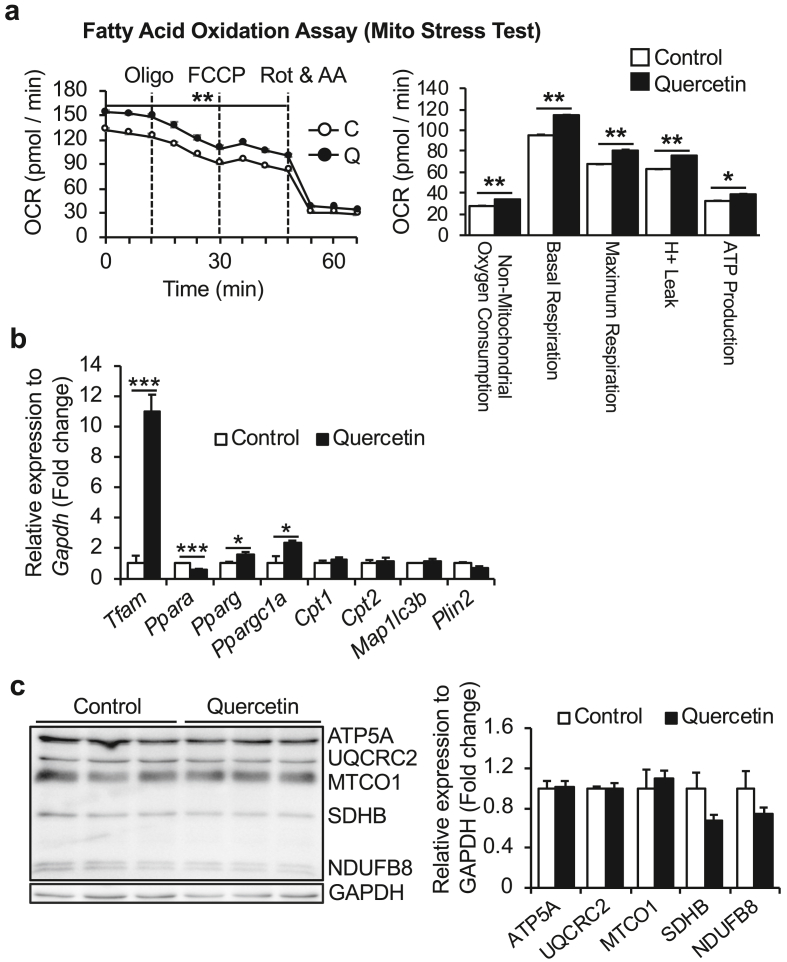
Effect of quercetin on fatty acid β-oxidation and mitochondrial marker expression of AML12 hepatocytes. (a) Fatty acid oxidation (FAO) assay results indicated enhancement of fatty acid β-oxidation by quercetin treatment (10 μM) for 12 h. FAO was measured by adding palmitate conjugated to BSA into XF base media, combined with Mito stress test (n = 3). (b) Expression of genes related to mitochondrial biogenesis, lipid metabolism, and autophagy (*Tfam, Ppara, Pparg, Ppargc1a, Cpt1, Cpt2, Map1lc3b*, and *Plin2*) were measured by qRT-PCR. mRNA expression levels were calculated relative to *Gapdh* and data are expressed as a fold-increase (n = 6). (c) Expression of mitochondrial oxidative phosphorylation proteins (ATP5A, UQCR2, MTCO1, SDHB, and NDUFB8) was measured by immunoblot (left). Protein expression levels were calculated relative to GAPDH. Right graphs show relative intensity of each band (n = 3). Values represent mean ± SEM. Significance was determined with a two-tailed Student's t-test (∗p < 0.05, ∗∗p < 0.01, ∗∗∗p < 0.001).

Although quercetin enhanced the mitochondrial respiration of AML12 hepatocytes, it was unclear whether this effect depended on enhanced fatty acid β-oxidation. To directly measure fatty acid β-oxidation, a palmitate-BSA FAO assay was performed in combination with the Mito Stress Test. In this assay, AML12 hepatocytes were cultured for 12 h in KB buffer containing L-carnitine (an essential dipeptide for mitochondrial inner membrane transport of acyl-coenzyme A) with or without quercetin, and the Mito Stress Test was performed to measure the OCR. Palmitate-BSA FAO assay results showed that quercetin significantly increased the OCR of AML12 hepatocytes, suggesting enhanced fatty acid β-oxidation (Figure 2a). At a molecular level, expression levels of mitochondrial biogenesis-related genes (*Tfam* and *Ppargc1a*) were significantly increased 12 h after quercetin treatment (Figure 2b). Although quercetin affected the expression levels of lipid metabolism-related genes (*Ppara* and *Pparg*), expression of genes for fatty acid transporters (*Cpt1, Cpt2*), lipid droplets (*Plin2*), and autophagy (*Map1lc3b*) was unaltered (Figure 2b). To determine whether enhanced mitochondrial respiration induced by quercetin treatment depended on mitochondrial oxidative phosphorylation (OXPHOS) proteins, their expression levels were measured by immunoblot. Quercetin did not affect OXPHOS protein expression in AML12 hepatocytes (Figure 2c), suggesting that the enhanced energy metabolism induced by quercetin may involve increased fatty acid β-oxidation and is at least partially independent of upregulation of mitochondrial biogenesis- and lipid metabolism-related genes.

### 3.3 Quercetin promoted fatty acid β-oxidation through lipophagy in AML12 hepatocytes

**Figure 3:**
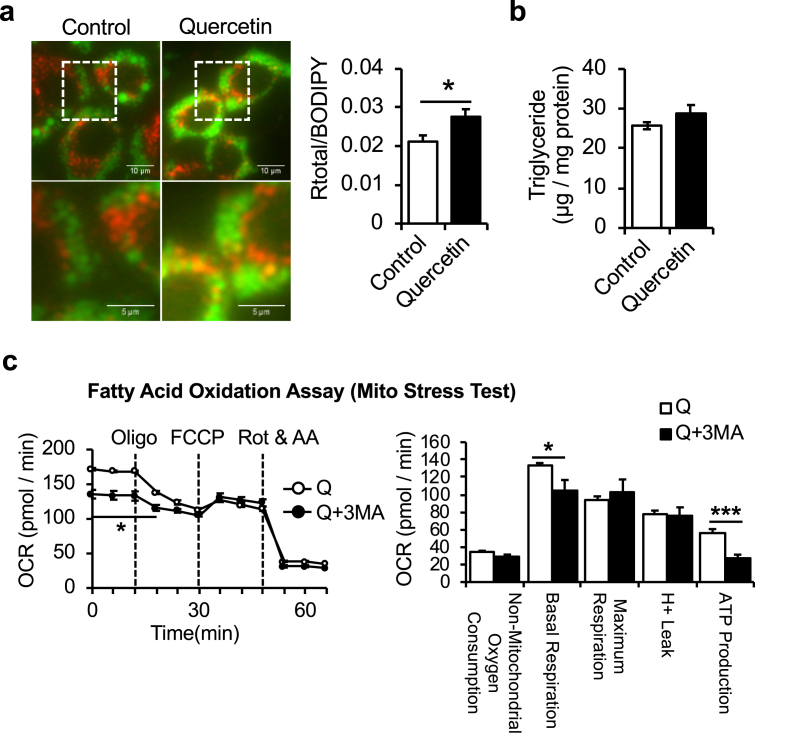
Relationship between quercetin and autophagy/lipophagy in AML12 hepatocytes. (a) Representative images of subcellular location of lipid droplets (BODIPY: green) and lysosomes (lysotracker: red) in AML12 hepatocytes treated with quercetin for 24 h (left: control, right: quercetin). Co-localization of BODIPY and lysotracker was evaluated by ImageJ co-localization analysis (n = 7). Scale bar = 10 μm. (b) Comparison of triglycerides between control and quercetin-treated (50 μM) AML12 hepatocytes (n = 6). (c) Fatty acid oxidation (FAO) assay showed that the enhancement fatty acid β-oxidation by quercetin treatment (10 μM) was cancelled by the autophagy–lysosomal pathway inhibitor 3-Methyladenine (3-MA). FAO was measured by adding palmitate conjugated to BSA into XF base media, combined with Mito stress test (n = 3). Values represent mean ± SEM. Significance was determined with a two-tailed Student's t-test (∗p < 0.05, ∗∗p < 0.01, ∗∗∗p < 0.001).

Autophagy contributes to the degradation of intracellular lipid droplets, termed “lipophagy”. Thus, we explored the possibility that lipophagy contributes to increased fatty acid β-oxidation induced by quercetin treatment. To visualize lipophagy in AML12 hepatocytes, lipid droplets and lysosomes were stained by BODIPY and LysoTracker Red, respectively (Figure 3a). Lipid droplets in AML12 hepatocytes treated with quercetin showed increased colocalization of BODIPY and LysoTracker Red fluorescence compared with controls, suggesting enhanced lipophagy. However, triglyceride contents were unaltered by quercetin treatment for 24 h (Figure 3b). To further obtain evidence of enhanced lipophagy by quercetin, the pharmacological autophagy–lysosomal pathway inhibitor 3-methyladenine (3-MA), was used in the palmitate-BSA FAO assay. As a result, inhibition of autophagy by 3-MA abolished the increase of fatty acid β-oxidation induced by quercetin in AML12 hepatocytes (Figure 3c), suggesting that enhanced lipophagy contributed to increased fatty acid β-oxidation.

### 3.4 Quercetin activated AMPK and ERK signaling in AML12 hepatocytes

**Figure 4:**
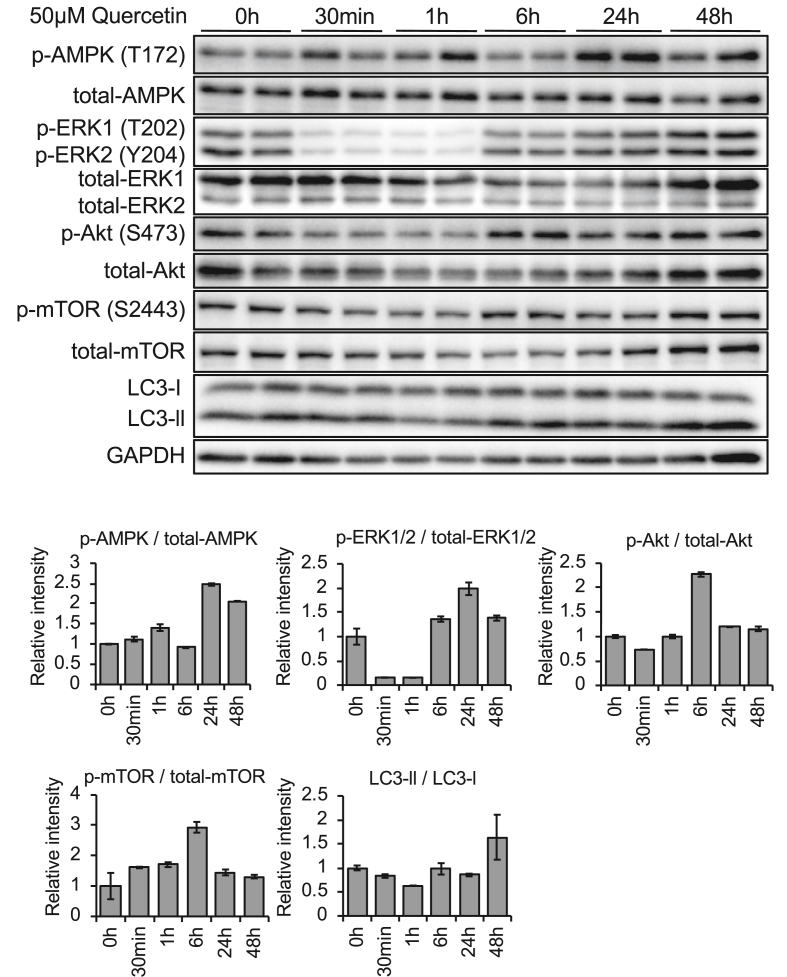
Effect of quercetin on autophagy-related signaling pathways in AML12 hepatocytes. The effect of quercetin (50 μM) on phosphorylation levels of AMPK (T172), ERK1/2 (T202 and Y204), Akt (S473), and mTOR (S2443), as well as activation of autophagy (ratio of LC3-II/LC3-I) were measured by immunoblot. Cells were cultured in serum-free DMEM/F-12 for 12 h before quercetin treatment. Phosphorylation levels were calculated relative to total protein (n = 3). Values represent mean ± SEM.

Regulation of lipophagy is not well understood, but its regulatory signaling pathways are likely associated with autophagy [24, 25]. Thus, we next investigated whether autophagy was induced by quercetin in AML12 hepatocytes under serum-free conditions. The activation level of autophagy was measured by the ratio of two forms of microtubule-associated protein light chain 3 (LC3-II/LC3-I), an autophagic membrane protein. Although quercetin did not alter mRNA expression of *Map1lc3b* (Figure 2b), a major autophagy marker, treatment with quercetin for 48 h increased the LC3-ll/LC3-l (translated product of *Map1lc3b*) ratio, suggesting activation of autophagy (Figure 4). Next, to investigate signaling pathways involved in activation of autophagy by quercetin in AML12 hepatocytes, phosphorylation levels of mTOR (a central regulator of autophagy) and its upstream and downstream targets Akt, adenosine monophosphate-activated protein kinase (AMPK), and extracellular signal regulated kinase 1/2 (ERK1/2) were measured by immunoblot. The results showed that 24-h quercetin treatment increased phosphorylation of both AMPK and ERK, suggesting that quercetin enhanced autophagy/lipophagy.

## 4 Discussion

Quercetin is a naturally occurring flavonoid with diverse biological activities including antioxidant, anticancer, anti-inflammatory, pro-apoptotic, and anti-diabetic properties [10]. As a novel biological activity, we recently reported that quercetin and isorhamnetin (a metabolite of quercetin in mammals) suppressed uric acid production in AML12 hepatocytes, as well as plasma and hepatic uric acid levels in a hyperuricemic model mice [26]. Although quercetin has reported hepatoprotective effects, whether it can directly enhance energy metabolism in hepatocytes was unclear. Thus, in the present study, we showed direct evidence that quercetin increased energy metabolism (mitochondrial respiration and glycolysis) through enhanced fatty acid β-oxidation in AML12 hepatocytes. Previous reports suggest that chronic supplementation of quercetin results in upregulated expression of genes related to lipid metabolism, mitochondrial biogenesis, and their transcriptional regulators [8, 9, 10]. As mentioned in the introduction, augmentation of gene expression by quercetin is a plausible mechanisms of its anti-obesity/diabetic properties. Indeed, the results of our qRT-PCR analysis revealed increased expression of mitochondrial biogenesis-related (*Tfam* and *Ppargc1a*) and lipid metabolism-related genes (*Ppara* and *Pparg*) in AML12 hepatocytes following quercetin treatment for 12 h. However, as expression of mitochondrial OXPHOS proteins, which are directly involved in mitochondrial oxygen consumption, were unaltered 12 h after quercetin treatment, we focused on a different mechanism to explain increased mitochondrial respiration in AML12 hepatocytes.

Recent reports demonstrated that chronic supplementation with quercetin (8–12 weeks) induced lipophagy, which involves the selective autophagic degradation of lipid droplets, in the livers of animal models of ALD and NAFLD [17, 18]; moreover, inhibited lipid accumulation and damage in the livers of these animals were attributed to this effect. However, it remained obscure whether the observed inhibition of lipid accumulation induced by quercetin depended on induction of lipophagy. By combining the autophagy inhibitor 3-MA with an FAO assay, we demonstrated that inhibition of autophagy abolished the enhancement of fatty acid β-oxidation induced by quercetin in AML12 hepatocytes. In addition, double staining of lipid droplets and lysosomes indicated the possibility that quercetin induced lipophagy in AML12 hepatocytes. As mentioned above, signaling pathways regulating lipophagy are not well understood, but may overlap with those regulating autophagy [24, 25]. The observed enhancement of mitochondrial respiration was likely caused by an acute (as opposed to chronic) effect of quercetin, such as immediate inactivation or activation of intracellular signaling pathways related to the regulation of autophagy. AMPK, a cellular energy sensor and regulator of autophagy [27], was activated 24 h after quercetin treatment in AML12 hepatocytes despite their nutrient-sufficient condition (serum-starved DMEM), suggesting the possibility of induced autophagy/lipophagy. Interestingly, ERK was inactivated within 30 min of quercetin treatment, which may be related to the anti-oxidant activity of quercetin because ERK is activated by reactive oxygen species.

Taken together, our results demonstrate that quercetin enhanced fatty acid β-oxidation by inducing lipophagy through AMPK signaling in AML12 hepatocytes. Moreover, our findings suggest a possible mechanism explaining the inhibitory effect of quercetin supplementation on lipid accumulation in the liver. However, we acknowledge the limitations of our study, such as the use of limited concentrations of quercetin (10–50 μM) that were non-physiological. As it was previously reported that a low dose of dietary quercetin (0.02%) for 16 weeks failed to attenuate the development of an obese phenotype in mice [28], we employed higher concentrations in this study. In addition, triglyceride levels were not altered following 24-h treatment with quercetin in AML12 hepatocytes. Although quercetin might acutely induce lipophagy/autophagy in AML12 hepatocytes, higher doses or chronic treatment of quercetin should be examined to assess the effects of quercetin on triglycerides. Moreover, whether a lower dose of quercetin could enhance fatty acid β-oxidation through induction of lipophagy in AML12 hepatocytes requires further investigation.

## Declarations

### Author contribution statement

Misato Fukaya: Performed the experiments; Analyzed and interpreted the data.

Yoriko Sato, Shinji Kondo, Shin-ichi Adachi, Fumiaki Yoshizawa: Contributed reagents, materials, analysis tools or data.

Yusuke Sato: Conceived and designed the experiments; Wrote the paper.

### Funding statement

This work was supported by 10.13039/501100012014Kieikai Research Foundation (2020S030) and partially supported by the Super Science High School Project of JST from the Ministry of Education, Science, Sports and Culture of Japan.

### Data availability statement

Data will be made available on request.

### Declaration of interests statement

The authors declare no conflict of interest.

### Additional information

No additional information is available for this paper.

## Appendix A Supplementary data

Supplemental Figure 1Uncropped immunoblot images of Figure 2C and Figure 4.

## References

[1] Erlund I. (2004). Review of the flavonoids quercetin, hesperetin, and naringenin. Dietary sources, bioactivities, bioavailability, and epidemiology. Nutr. Res..

[2] Formica J.V., Regelson W. (1995). Review of the biology of Quercetin and related bioflavonoids. Food Chem. Toxicol..

[3] Zhao Y., Chen B., Shen J., Wan L., Zhu Y., Yi T., Xiao Z. (2017). The beneficial effects of quercetin, curcumin, and resveratrol in obesity. Oxid. Med. Cell. Longev.

[4] Anand David A.V., Arulmoli R., Parasuraman S. (2016). Overviews of biological importance of quercetin: a bioactive flavonoid. Pharmacogn. Rev..

[5] Lee J.S., Cha Y.J., Lee K.H., Yim J.E. (2016). Onion peel extract reduces the percentage of body fat in overweight and obese subjects: a 12-week, randomized, double-blind, placebo-controlled study. Nutr. Res. Pract..

[6] Kim K.A., Yim J.E. (2016). The effect of onion peel extract on inflammatory mediators in Korean overweight and obese women. Clin. Nutr. Res..

[7] Panchal S.K., Poudyal H., Brown L. (2012). Quercetin ameliorates cardiovascular, hepatic, and metabolic changes in diet-induced metabolic syndrome in rats. J. Nutr..

[8] Kobori M., Masumoto S., Akimoto Y., Takahashi Y. (2009). Dietary quercetin alleviates diabetic symptoms and reduces streptozotocin-induced disturbance of hepatic gene expression in mice. Mol. Nutr. Food Res..

[9] Kobori M., Masumoto S., Akimoto Y., Oike H. (2011). Chronic dietary intake of quercetin alleviates hepatic fat accumulation associated with consumption of a Western-style diet in C57/BL6J mice. Mol. Nutr. Food Res..

[10] Kobori M., Takahashi Y., Sakurai M., Akimoto Y., Tsushida T., Oike H., Ippoushi K. (2016). Quercetin suppresses immune cell accumulation and improves mitochondrial gene expression in adipose tissue of diet-induced obese mice. Mol. Nutr. Food Res..

[11] Li X., Wang R., Zhou N., Wang X., Liu Q., Bai Y., Bai Y., Liu Z., Yang H., Zou J., Wang H., Shi T. (2013). Quercetin improves insulin resistance and hepatic lipid accumulation in vitro in a NAFLD cell model. Biomed. Rep..

[12] Porras D., Nistal E., Martinez-Florez S., Pisonero-Vaquero S., Olcoz J.L., Jover R., Gonzalez-Gallego J., Garcia-Mediavilla M.V., Sanchez-Campos S. (2017). Protective effect of quercetin on high-fat diet-induced non-alcoholic fatty liver disease in mice is mediated by modulating intestinal microbiota imbalance and related gut-liver axis activation. Free Radic. Biol. Med..

[13] Zhu M., Zhou X., Zhao J. (2017). Quercetin prevents alcohol-induced liver injury through targeting of PI3K/Akt/nuclear factor-kappaB and STAT3 signaling pathway. Exp. Ther. Med..

[14] Yao P., Nussler A., Liu L., Hao L., Song F., Schirmeier A., Nussler N. (2007). Quercetin protects human hepatocytes from ethanol-derived oxidative stress by inducing heme oxygenase-1 via the MAPK/Nrf 2 pathways. J. Hepatol..

[15] Kim C.S., Choi H.S., Joe Y., Chung H.T., Yu R. (2016). Induction of heme oxygenase-1 with dietary quercetin reduces obesity-induced hepatic inflammation through macrophage phenotype switching. Nutr. Res. Pract..

[16] Lee Y.J., Beak S.Y., Choi I., Sung J.S. (2018). Quercetin and its metabolites protect hepatocytes against ethanol-induced oxidative stress by activation of Nrf 2 and AP-1. Food Sci. Biotechnol..

[17] Zhu X., Xiong T., Liu P., Guo X., Xiao L., Zhou F., Tang Y., Yao P. (2018). Quercetin ameliorates HFD-induced NAFLD by promoting hepatic VLDL assembly and lipophagy via the IRE1a/XBP1s pathway. Food Chem. Toxicol..

[18] Zeng H., Guo X., Zhou F., Xiao L., Liu J., Jiang C., Xing M., Yao P. (2019). Quercetin alleviates ethanol-induced liver steatosis associated with improvement of lipophagy. Food Chem. Toxicol..

[19] Wang K., Liu R., Li J., Mao J., Lei Y., Wu J., Zeng J., Zhang T., Wu H., Chen L., Huang C., Wei Y. (2011). Quercetin induces protective autophagy in gastric cancer cells: involvement of Akt-mTOR- and hypoxia-induced factor 1 alpha-mediated signaling. Autophagy.

[20] Granato M., Rizzello C., Gilardini Montani M.S., Cuomo L., Vitillo M., Santarelli R., Gonnella R., D'Orazi G., Faggioni A., Cirone M. (2017). Quercetin induces apoptosis and autophagy in primary effusion lymphoma cells by inhibiting PI3K/AKT/mTOR and STAT3 signaling pathways. J. Nutr. Biochem..

[21] Liu Y., Gong W., Yang Z.Y., Zhou X.S., Gong C., Zhang T.R., Wei X., Ma D., Ye F., Gao Q.L. (2017). Quercetin induces protective autophagy and apoptosis through ER stress via the p-STAT3/Bcl-2 axis in ovarian cancer. Apoptosis.

[22] Jung C.H., Ro S.H., Cao J., Otto N.M., Kim D.H. (2010). mTOR regulation of autophagy. FEBS Lett..

[23] Han J., Wang Y. (2018). mTORC1 signaling in hepatic lipid metabolism. Protein Cell.

[24] Ward C., Martinez-Lopez N., Otten E.G., Carroll B., Maetzel D., Singh R., Sarkar S., Korolchuk V.I. (2016). Autophagy, lipophagy and lysosomal lipid storage disorders. Biochim. Biophys. Acta.

[25] Zechner R., Madeo F., Kratky D. (2017). Cytosolic lipolysis and lipophagy: two sides of the same coin. Nat. Rev. Mol. Cell Biol..

[26] Adachi S.I., Kondo S., Sato Y., Yoshizawa F., Yagasaki K. (2019). Anti-hyperuricemic effect of isorhamnetin in cultured hepatocytes and model mice: structure-activity relationships of methylquercetins as inhibitors of uric acid production. Cytotechnology.

[27] Kim J., Kundu M., Viollet B., Guan K.L. (2011). AMPK and mTOR regulate autophagy through direct phosphorylation of Ulk1. Nat. Cell Biol..

[28] Enos R.T., Velazquez K.T., Carson M.S., McClellan J.L., Nagarkatti P., Nagarkatti M., Davis J.M., Murphy E.A. (2016). A low dose of dietary quercetin fails to protect against the development of an obese phenotype in mice. PloS One.

