# Supplementary material for: Quercetin enhances fatty acid β-oxidation by inducing lipophagy in AML12 hepatocytes
Source: Heliyon. 2021 Jun 18;7(6):e07324. doi: 10.1016/j.heliyon.2021.e07324 (PMC8233147; doi:10.1016/j.heliyon.2021.e07324)
Supplement: Supplementary file 1 — Supplemental Figure 1Uncropped immunoblot images of Figure 2C and Figure 4. [file mmc1.pptx]

## Slide 1
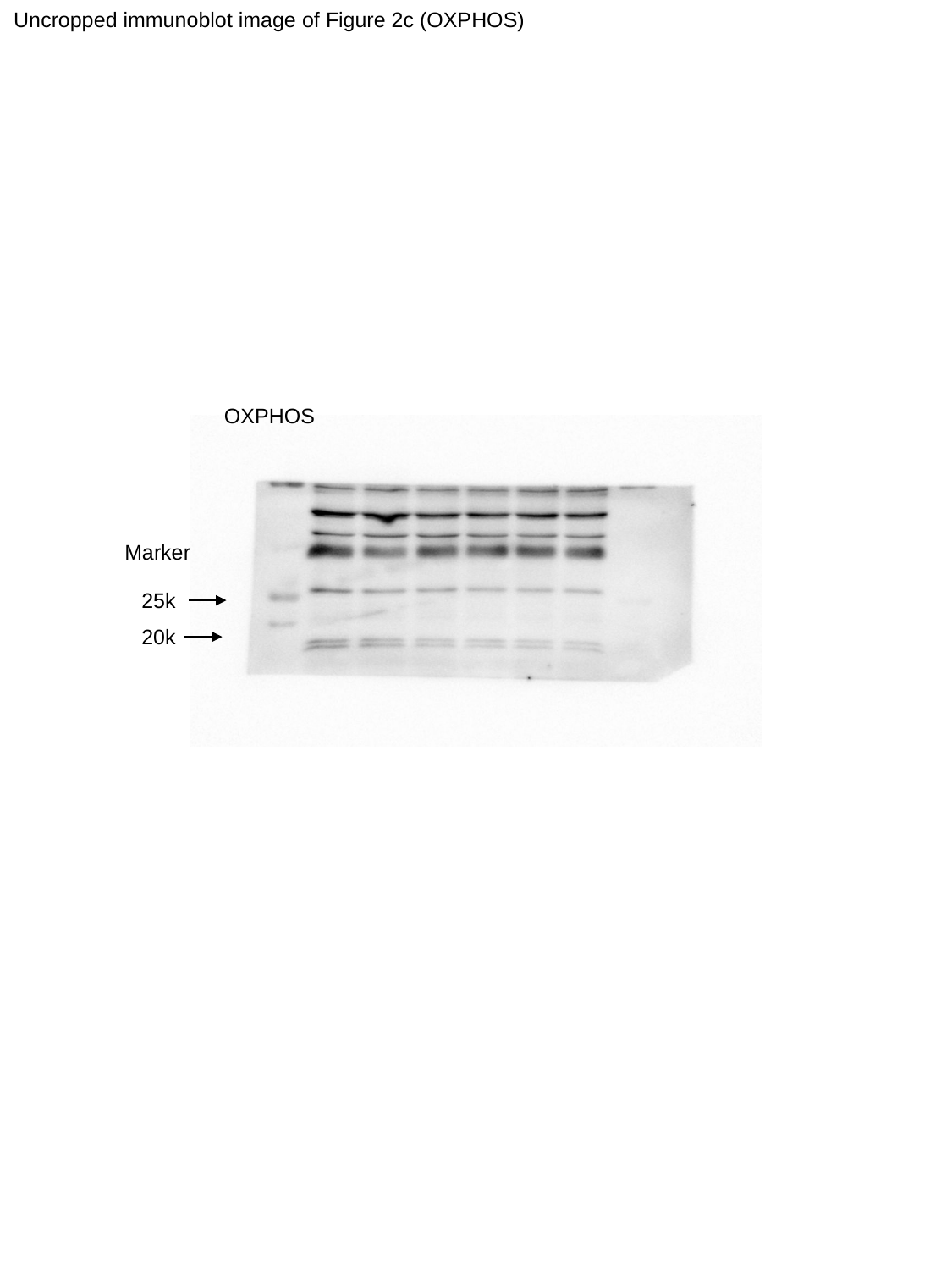

Uncropped immunoblot image of Figure 2c (OXPHOS)
OXPHOS
Marker
25k
20k

## Slide 2
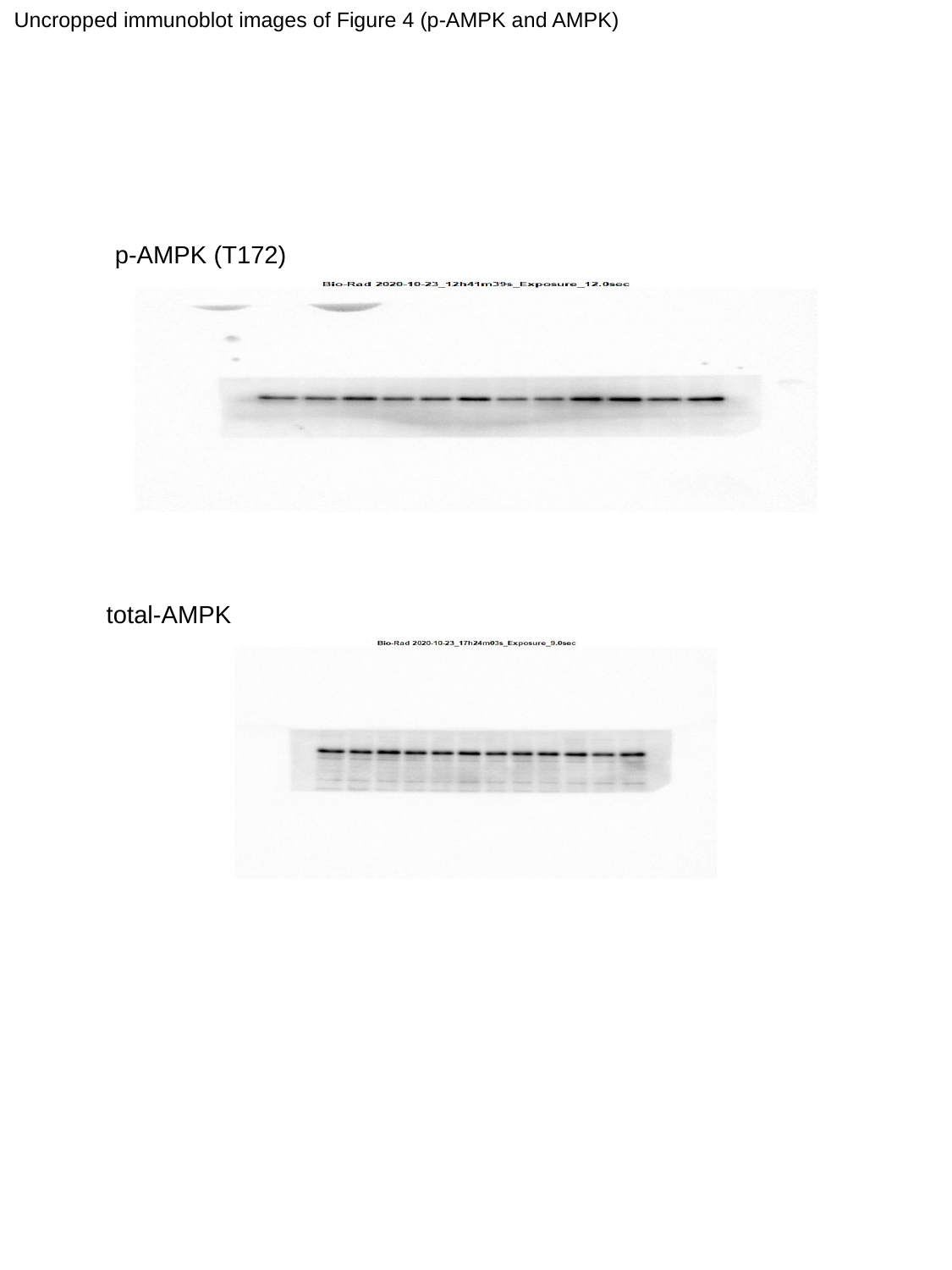

Uncropped immunoblot images of Figure 4 (p-AMPK and AMPK)
p-AMPK (T172)
total-AMPK

## Slide 3
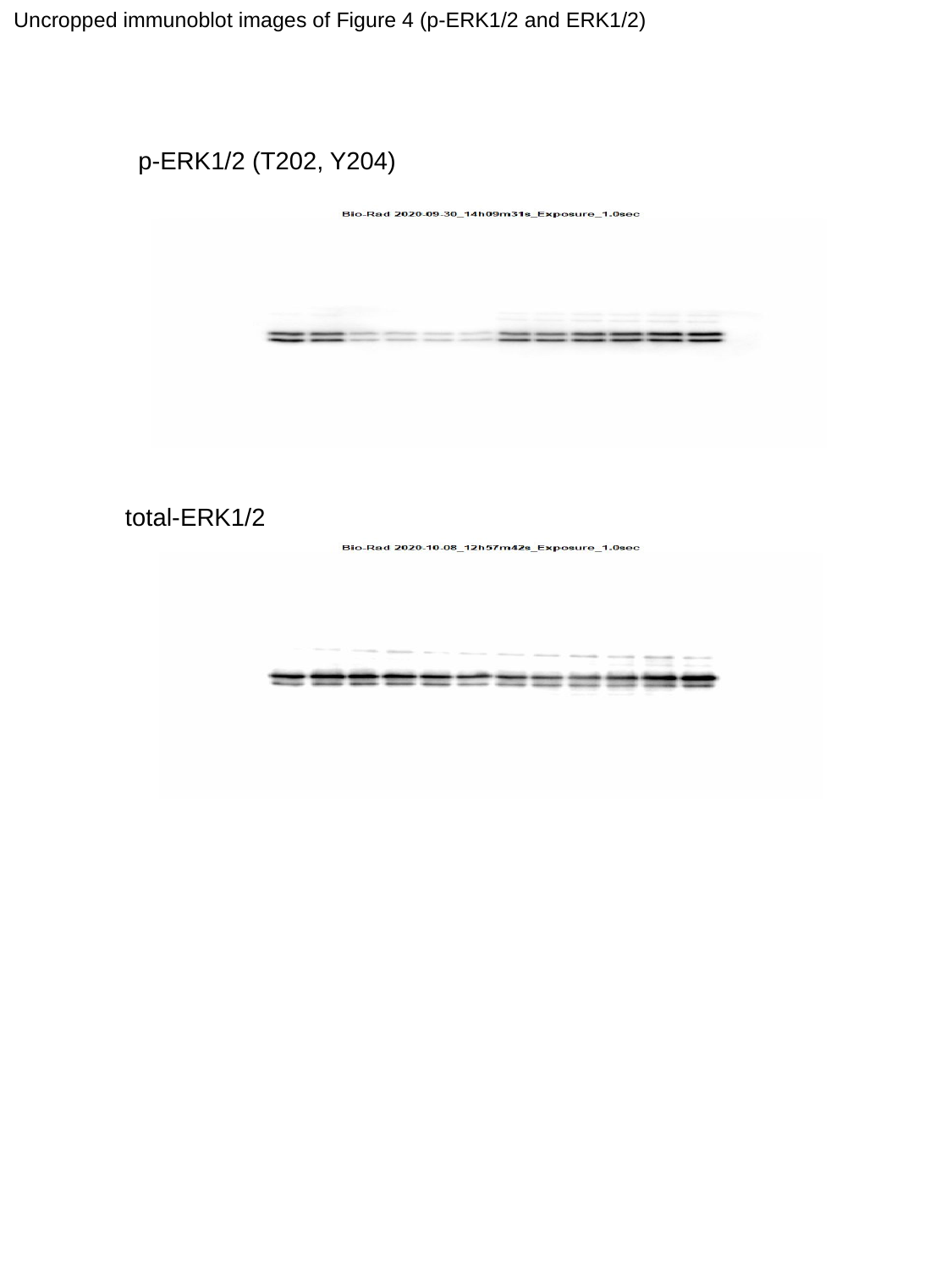

Uncropped immunoblot images of Figure 4 (p-ERK1/2 and ERK1/2)
p-ERK1/2 (T202, Y204)
total-ERK1/2

## Slide 4
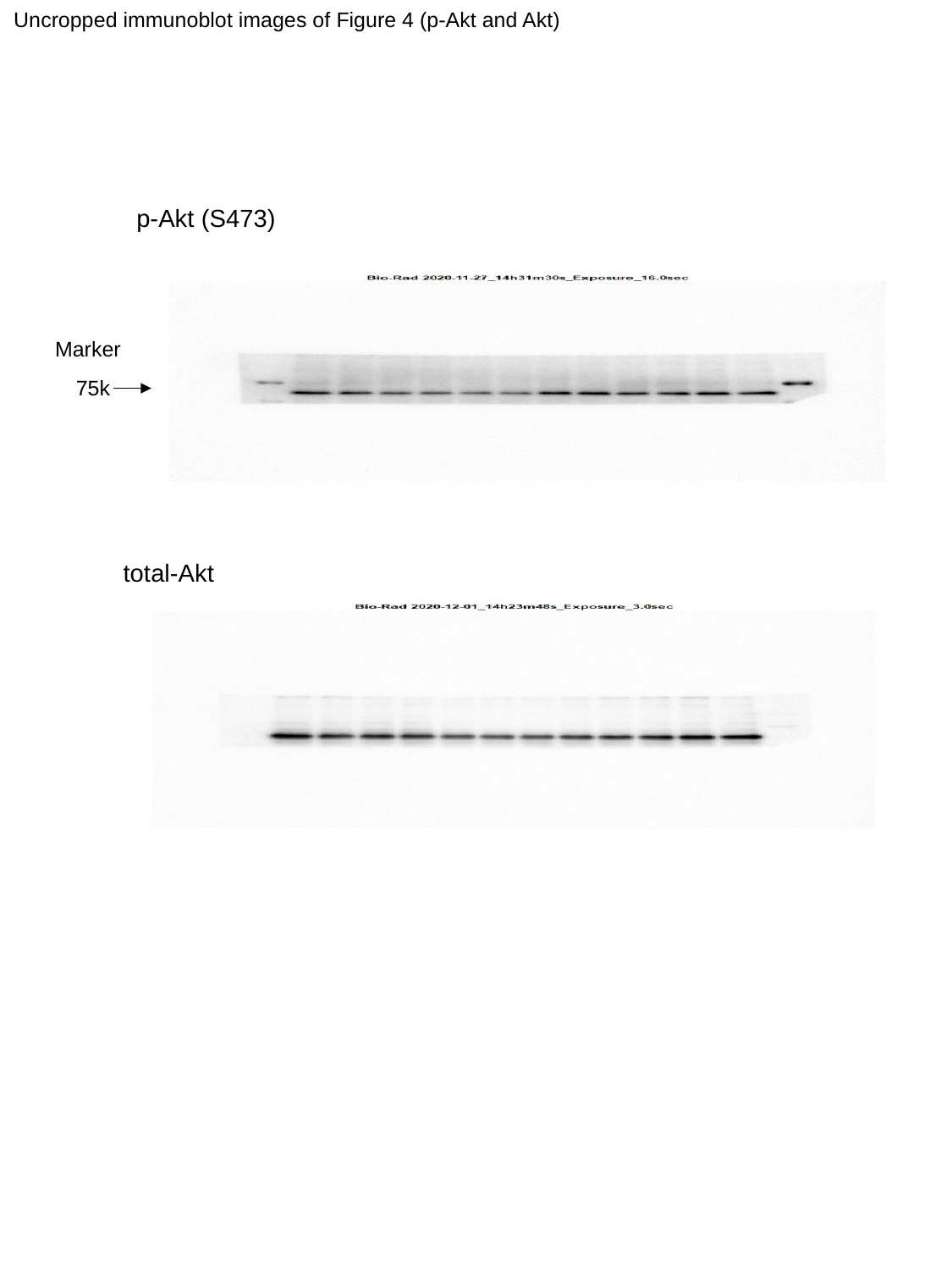

Uncropped immunoblot images of Figure 4 (p-Akt and Akt)
p-Akt (S473)
Marker
75k
total-Akt

## Slide 5
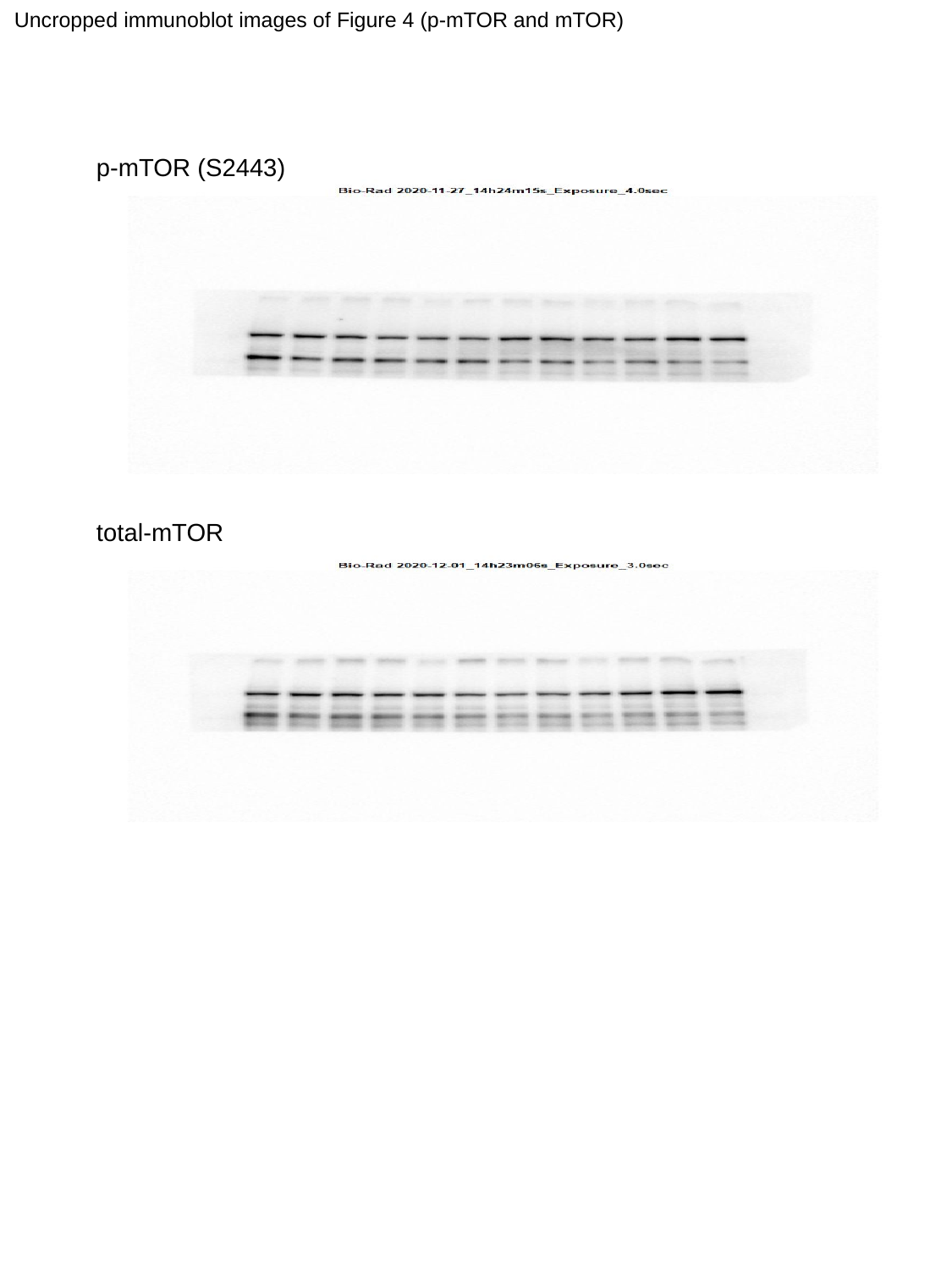

Uncropped immunoblot images of Figure 4 (p-mTOR and mTOR)
p-mTOR (S2443)
total-mTOR

## Slide 6
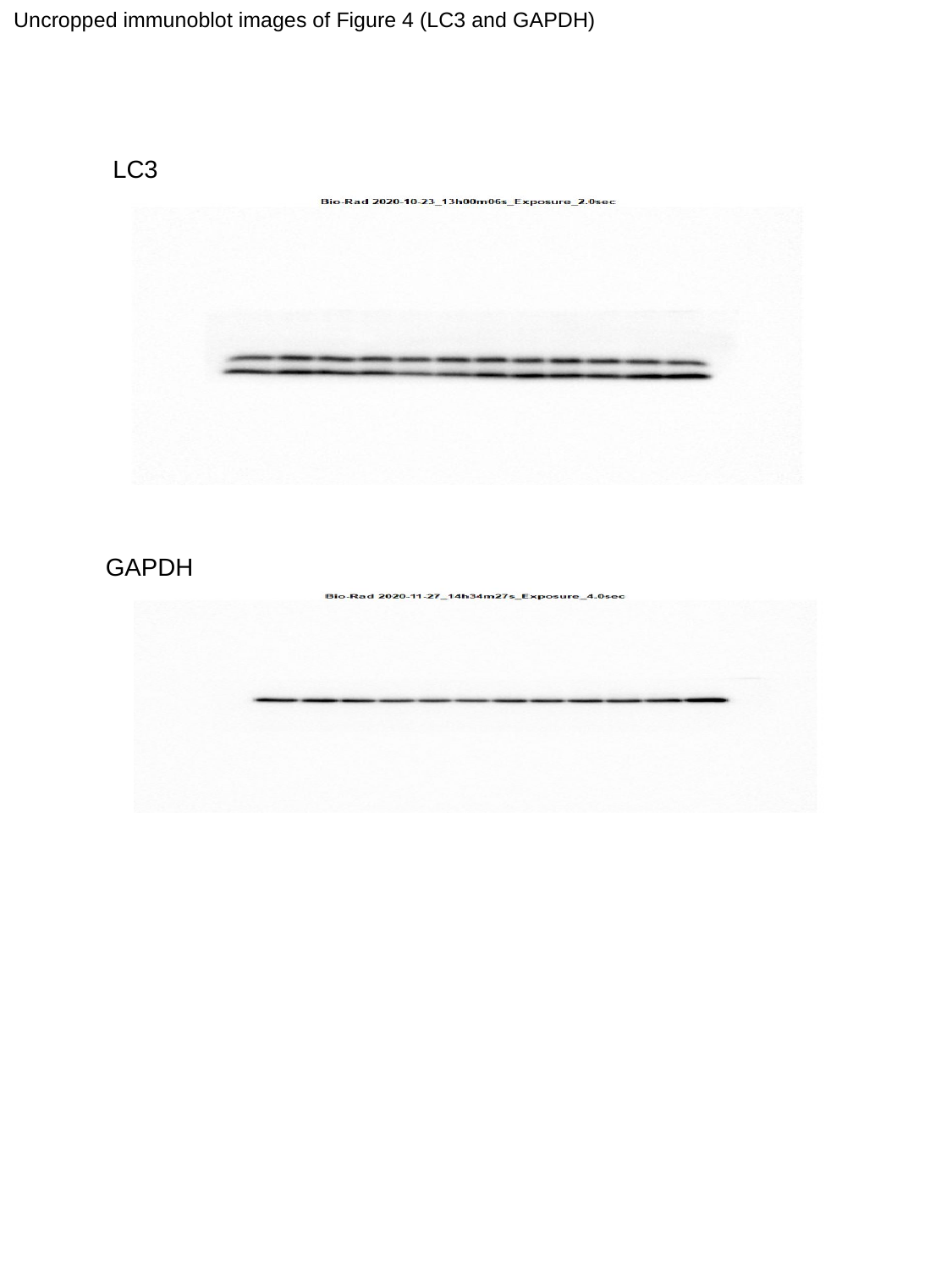

Uncropped immunoblot images of Figure 4 (LC3 and GAPDH)
LC3
GAPDH
